# Supplementary figures and images for: Exploring canine’s olfactive threshold in artificial urine for medical detection
Source: PLoS One. 2025 May 2;20(5):e0321394. doi: 10.1371/journal.pone.0321394 (PMC12047819; doi:10.1371/journal.pone.0321394)

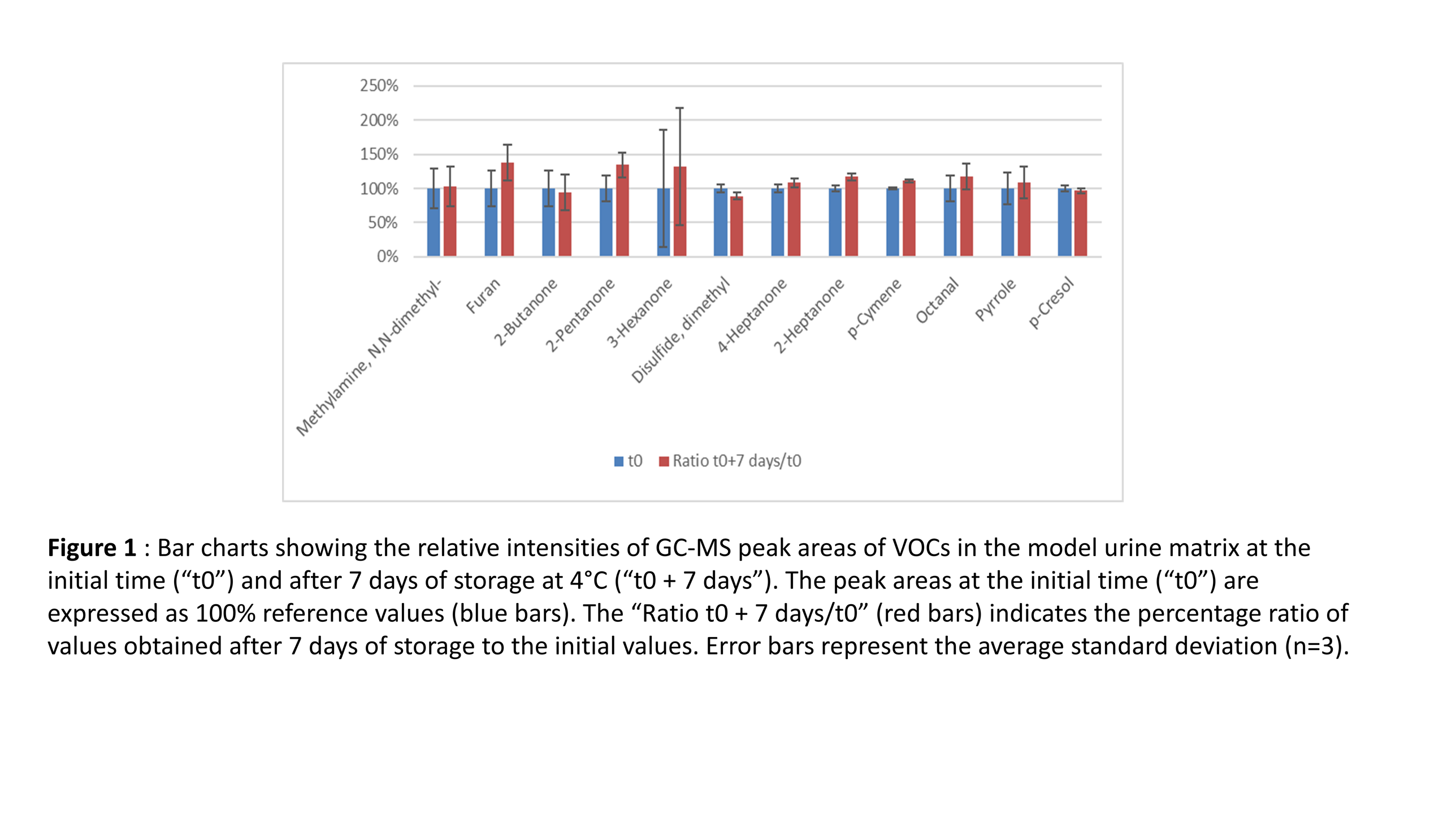

Supplement: S1 Fig — (TIF) [file pone.0321394.s005.TIF]

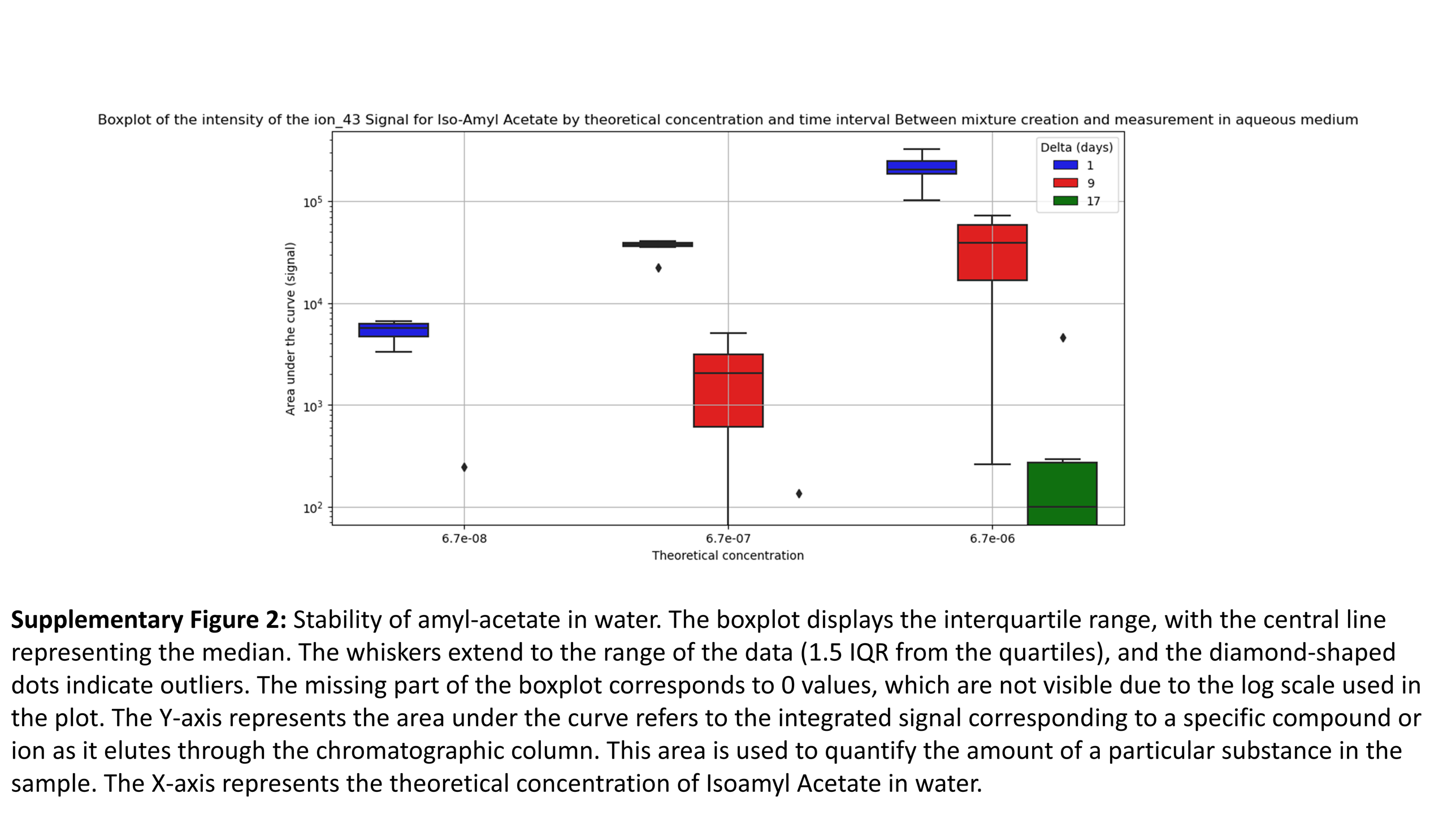

Supplement: S2 Fig — (TIF) [file pone.0321394.s006.TIF]

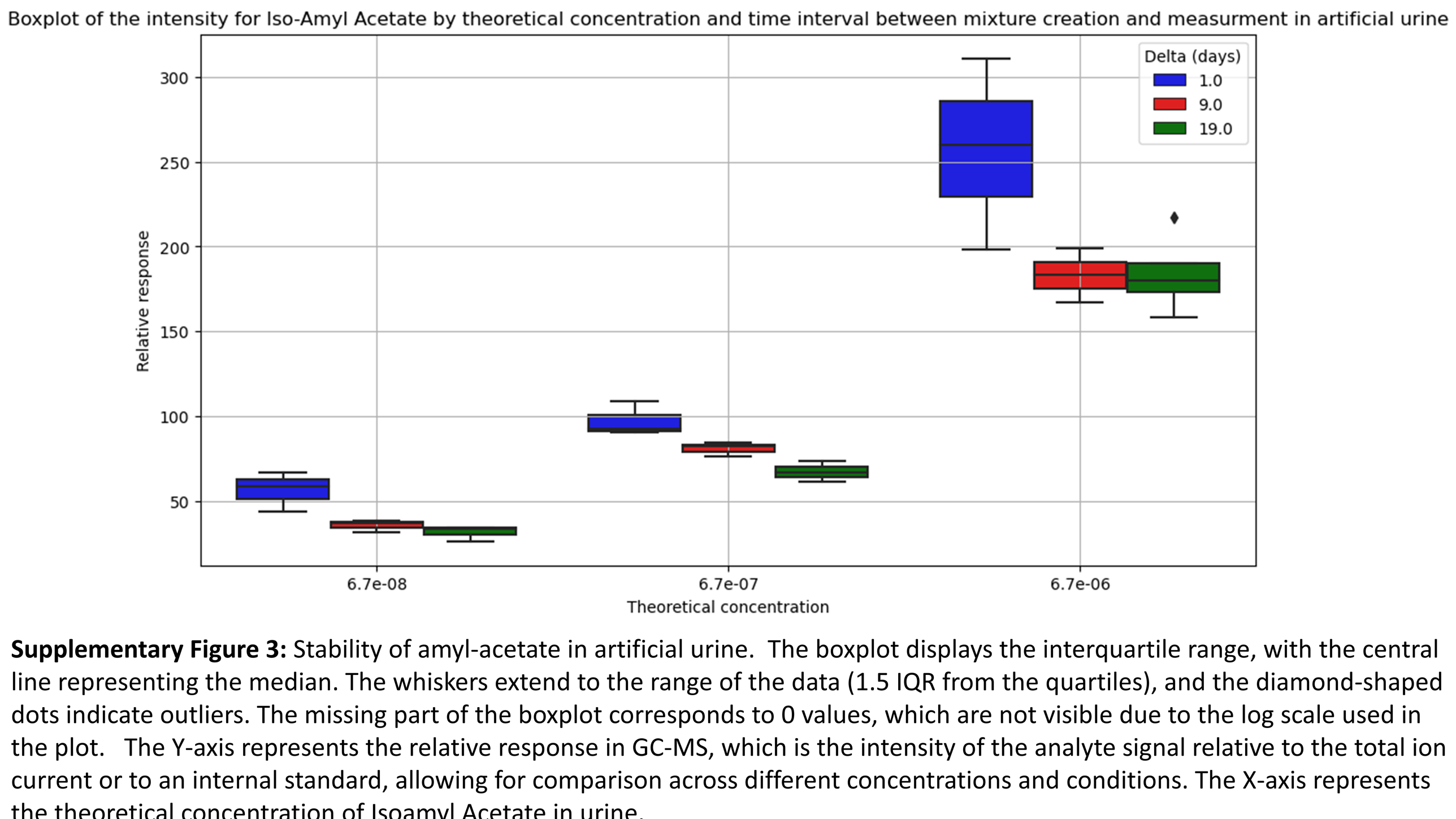

Supplement: S3 Fig — (TIF) [file pone.0321394.s007.TIF]
