# Supplementary material for: Exploring canine’s olfactive threshold in artificial urine for medical detection
Source: PLoS One. 2025 May 2;20(5):e0321394. doi: 10.1371/journal.pone.0321394 (PMC12047819; doi:10.1371/journal.pone.0321394)
Supplement: Attachment — Submitted filename: Response_to_reviewers_P3.docx [file pone.0321394.s009.docx]

Dear,

Thank you for the constructive comments and suggestions. We have revised the manuscript in accordance with the reviewers' recommendations. Below, you will find our detailed responses to each comment. Additionally, we have attached an updated version of the manuscript with track changes highlighting the modifications made in response to each reviewer’s feedback.

Kind regards,

Michelle Leemans

Reviewers' comments:

Reviewer's Responses to Questions

**Comments to the Author**

1. Is the manuscript technically sound, and do the data support the conclusions?

The manuscript must describe a technically sound piece of scientific research with data that supports the conclusions. Experiments must have been conducted rigorously, with appropriate controls, replication, and sample sizes. The conclusions must be drawn appropriately based on the data presented.

Reviewer #1: Partly

Reviewer #2: Partly

2. Has the statistical analysis been performed appropriately and rigorously?

Reviewer #1: Yes

Reviewer #2: Yes

3. Have the authors made all data underlying the findings in their manuscript fully available?

The [PLOS Data policy](http://track.editorialmanager.com/CL0/http:%2F%2Fwww.plosone.org%2Fstatic%2Fpolicies.action%23sharing/1/010f0193bf50fa05-6bc64c74-d5ab-4d63-a44e-b1b3571f2d93-000000/qmBBHsfndLSDbEAxzj_mkGDrSTEuuZuZfF6rYancurc=189) requires authors to make all data underlying the findings described in their manuscript fully available without restriction, with rare exception (please refer to the Data Availability Statement in the manuscript PDF file). The data should be provided as part of the manuscript or its supporting information, or deposited to a public repository. For example, in addition to summary statistics, the data points behind means, medians and variance measures should be available. If there are restrictions on publicly sharing data—e.g. participant privacy or use of data from a third party—those must be specified.

Reviewer #1: Yes

Reviewer #2: No

4. Is the manuscript presented in an intelligible fashion and written in standard English?

PLOS ONE does not copyedit accepted manuscripts, so the language in submitted articles must be clear, correct, and unambiguous. Any typographical or grammatical errors should be corrected at revision, so please note any specific errors here.

Reviewer #1: Yes

Reviewer #2: Yes

5. Review Comments to the Author

Please use the space provided to explain your answers to the questions above. You may also include additional comments for the author, including concerns about dual publication, research ethics, or publication ethics. (Please upload your review as an attachment if it exceeds 20,000 characters)

# Reviewer #1:

We need more data papers on working dogs and this very small - really a pilot study - can be a part of those data. The paper needs some work.

**1.1** Lines 125 – 126 – Please take out ‘being the first’ – you have 2 dogs with widely different performances – you an say nothing about generality. Just say “We trained 2 dogs….et cetera…”. Really, this should more appropriately be called a pilot study.

Line 142: Thank you for your comment. We have revised the sentence. We also acknowledge that this is a pilot study and will make this clearer in the manuscript.

**1.2** Lines 138-139 This is an inadequate description of their training – be specific.

Line 330: The description of the training and testing procedures has been moved to a more appropriate section of the manuscript, where it is presented in greater detail for better clarity and context.

**1.3** Line 142 I don’t know what the KDOG centre is and most readers will not either

The reference to the KDOG centre has been removed from the manuscript to avoid confusion.

**1.4** Line 239 “Motivation” is likely the wrong word here – you were assessing a dog’s interest (and many detection dog guidelines make this clear) the compound. There are a number of criteria used to evaluate motivation and you meet none of them. Please change wording throughout.

Line 386,389, 399, 500: We agree with the reviewer. The word "motivation" was primarily used by handlers, and we have continued to use this term, which is not appropriate. We have replaced "motivation" with "interest" throughout the manuscript.

**1.5** Line 271, 274 et cetera – ‘Frustration’ is another one of those words that is overly used and seldom defined. You did not reward them each time to decrease ‘frustration’ – however you define this. You did so because when you cannot know if the compound is there (eg, accelerants) you use a continuous reward schedule. You used this for 2 reasons: because you could not know in that phase (and this is why training where someone tells you in a double blind that you were correct is so critical – something that was apparently not done here and I cannot fathom why not) AND you were rewarding the dogs for working – for making a choice. You may want to read and cite some of the work of Adee Schoon on methodology for detection dogs.

We thank the reviewer for these comments, and we have removed the term frustration. Additionally, a section addressing the limitations has been added to the discussion.” *In future studies, an alternative training methodology, as recommended by Edwards et al., 2017, should be implemented. Specifically, the observer with information on the sample status could be positioned outside the room, equipped with a numerical screen to immediately respond to the trainer regarding the sample status. Indeed, occasional reinforcement of a positive indication for a disease-negative sample may lead to persistent false indications, as potentially observed in this study. This additional training step should be incorporated to validate the findings of this preliminary study.*”

**1.6** Line 291 – These authors did not create this ethogram to assess stress. Instead, this was an exhaustive ethogram of behaviors exhibited under a set of 3 request conditions. Working dogs dropped many of these out compared to pet dogs. Please correct the wording.

We thank the reviewer for this correction. We have adjusted the wording based on your feedback. The document is now titled "List of Behaviors Assessed by Video."

**1.7** Line 300 – Why don’t you define sensitivity and specificity first. Many people get it wrong.

Line 452: Thank you for your suggestion. We have now added definitions for sensitivity and specificity earlier in the manuscript to ensure clarity and to avoid potential confusion for readers.

**1.8** Line 332 – Again – remove motivation and use ‘interest’.

Line 386,389, 399, 500: The word motivation has been replaced by interest.

**1.9** Lines 349-350 – It’s not statistically significant. STOP HERE. You have 2 dogs. It’s not biologically meaningful to them or any subject who would hope to benefit from their efforts.

Thank you for your comment. We have removed the phrase as suggested.

**1.10** Lines 383+ - I am a big fan of a good literature review – but you had 2 dogs. You can talk about what other authors have opined but there is no relevance of any of this

Based on your suggestion, we have revised the text to focus more on the limitations of our study and avoid over-interpretation based on existing literature throughout the whole manuscript.

**1.11** Lines 385 + - Your observed differences highlight the need for an adequate sample size.

Line 697: Thank you for your comment. We have revised the manuscript to acknowledge that the observed performance differences highlight the need for a larger sample size to draw more meaningful conclusions. This revision emphasizes the importance of increasing the number of dogs in future studies to better understand the factors influencing canine medical detection.

**1.12** Lines 404+ - Here’s where commentary by Schoon and Jezierski could really help you.

Line 563, 567: It has indeed been demonstrated that if handlers have information about the responses of the protocol, this could lead to significant changes in the search behaviour of the dogs. We have added the reference to DeChant et al 2020 to the manuscript.

**1.13** Line 415 – Circular set up ref??

Line 710: A reference has been added in the updated version of the manuscript.

**1.14** Lines 435 + - This a very lengthy discussion for what is essentially a pilot study – you do not need most of this opinion about protocols et cetera. Condense all of this to the essentials.

Thank you for your feedback. We have significantly condensed the discussion, reducing its length by nearly half to emphasize the key findings while omitting unnecessary details about protocols.

**1.15** Also - please check that formatting and language are correct throughout for an English language journal. For example - quotes should be on "top".

We reviewed and corrected the formatting and language, ensuring consistency with English-language journal standards.

# Reviewer #2:

General comments:
This study examined two dogs' olfactory detection threshold for isoamyl acetate, which have been studied earlier. However, the authors created a more practical/lifelike situation by creating a stable artificial urine solution (which is a great achievement) and tested to what extent these two detection dogs were able to identify the isoamyl acetate in it.
**2.1** However, there is one major problem with the study. There is no difference between the single-blind and double-blind conditions. The authors stated that in single-blind tests: „The handler was unaware of the location of the positive sample, the position of the positive sample is known only to the operator in the adjacent building unit.” Then they state in the discussion: “In a single-blind situation, the handler, being blind, is the one who can provide clues that may subtly affect the dog's behavior. Double-blind protocols, while more complex to execute, provided additional assurance by eliminating potential biases from human handlers, thereby reinforcing the study's methodological integrity.”
In single-blind protocols only the operator knows the location of the target, but the operator is present in the test location (See Script 3 of Experiment 2 (page 3) in DeChant et al. (2020)! In this case the operator or the dog may be able to read subtle cues from the operator (not the handler who has no idea about the target location!) about the location of the target scent.
In double-blind protocols, neither the handler, nor the operator knows the location of the target scent, so the dog or the handler would not be able to read subtle cues about the location of the target from the operator. So by eliminating the presence of the operator next to the line-up during the single-blind protocol, the authors effectively made the situation double-blind.
Based on this the paper needs a major rewrite, in a way that the single-blind tests are either left out or merged with the double-blind test data and then a reanalysis is also needed.

Line 390-404: Thank you for your feedback. Indeed, the description of the single-blind and double-blind conditions was insufficient. To clarify, there is a window between the two units that allows the operator to communicate with the handler. This setup enables the handler to observe the operator's cues and reward the dog correctly when it makes the right decision, or withhold rewards if the dog makes an incorrect decision. We will revise the manuscript to include this important detail and to address the potential influence of such cues on the dog's behavior.

It is essential to note that if someone knows the results, biases can arise because dogs are remarkably adept at deciphering subtle cues. For example, Nougaro, one of the dogs, was able to interpret labels correctly. We encountered numerous instances where the intelligence of the dogs surprised us, emphasizing the need for extreme caution to avoid unintentional signaling. This is akin to the case of Hans, the "clever horse," which serves as a classic example of how animals can respond to subtle, unconscious cues from humans. With these considerations, we took meticulous care to minimize biases and ensure the integrity of the testing conditions.

**2.2** Apart from the above, the introduction and discussion are relatively well written, but the methods and results need clarifications. The tables and graphs also need further clarifications.

***Other specific comments:***

**2.3** L78: olfactory threshold OF dogs

Line 78: Thank you for your comment. The phrase "olfactory threshold OF dogs" has been corrected in the manuscript.

**2.4** L81-83: This should be rephrased as it sounds that the dogs were separately trained to indicate each different concentration of isoamyl acetate. However, I presume the dogs were trained to indicate the presence of isoamyl acetate.

Line 81-83: Thank you for your feedback. You are correct that the phrasing could be misleading. We have rephrased the sentence.

**2.5** L83: „consistent handlers” – consistent in what way?

Line 84: Thank you for your comment. To clarify, by "consistent handlers," we mean that the same two handlers were used throughout the study, ensuring a similar methodology and environment for all training and testing. We have revised the text.

**2.6** L84-85: „During double-blinded tests, handlers were unaware of the target’s position.” – this is common knowledge, so it can be deleted

Line 90: We have removed the statement "During double-blinded tests, handlers were unaware of the target’s position.”.

**2.7** L86: „Be consistent in the use of comma or dots for decimals. Use the one required by the journal.”; Do the dogs not have an ID number or names? Why are they just called one dog and the other?

Line 91: We have ensured consistency in the use of decimals. Regarding the dogs, we will add their names for clarity and consistency, rather than referring to them simply as "one dog" and "the other."

**2.8** L109-111: This is all true, however, it is important to bear in mind that a dog can work a couple of hours before it needs to rest. The amount of dogs that would be necessary for a cancer screening programme can be enormous. Covid detection dogs had the same hype, and this was one of the reasons, why they were not utilized in large scale, whether it be hospitals or public places.

Line 130: Thank you for your comment. We agree with the points raised regarding the challenges of using dogs for large-scale detection programs, such as the high number of dogs required for cancer screening. Additionally, to address this limitation, we are considering incorporating electronic noses alongside dogs to enhance detection capacity and improve scalability. We have added this consideration to the paragraph.

**2.9** L126: What is the relevance of isoamyl acetate from a medical point of view? Why did not the authors use some molecule that is medically relevant for cancer detection? What is the reason why many other studies used isoamyl acetate for olfactory detection threshold?

Line 243: Isoamyl acetate has no direct medical relevance but is widely used in olfactory research. We use it as a comparator to assess how dogs perform in complex vs. single mediums. Additionally, it has a distinct banana-like odor and is non-toxic. This explanation has been added to the manuscript. As a side note: We currently do not know which COV is really of disease-relevance and whether it is a single COV or a combination of multiple COVS.

**2.10** L131-132: This last sentence would fit more in the conclusion and not in the aim of the study.

The phrase has been removed from the introduction.

**2.11** L137: „were intact” – is already mentioned in the previous sentence

The duplication has been removed.

**2.12** L139-140: „They were exercised five days a week, receiving twice-daily off-leash walks in groups with other dogs and training sessions.” – This sentence is confusing, should be rephrased. Clarify how many training sessions and walks were done daily.

Line 336: They were exercised five days a week, receiving twice-daily off-leash walks in groups with alongside other dogs. Dogs participated in up to two sessions per day, one conducted in the morning before lunch and another in the afternoon. The sentence has been adapted in the updated version of the manuscript.

**2.13** L141: delete comma before „4300”

The comma has been deleted.

**2.14** L142: duplicate bracket

The duplicated bracket has been removed.

**2.15** L143: what was the temperature and humidity setting in the experimental room?

Line 340: Experiment room temperature averaged 20°C and humidity between 40% and 60%. This information has been added in the updated version of the manuscript.

**2.16** L143-144: „working with the same handler.” – It is mentioned above that the training was conducted by two experimenters, so please clarify.

Line 336: Each dog was assigned a specific handler and operator, and this clarification has been incorporated into the text.

**2.17** L162: Dot is missing from the end of the sentence. Why is the Furan spelled with a capital F, while the other compounds are not?

Line 205: The missing dot has been added, and "Furan" has been adapted to lowercase "f".

**2.18** L166: The year should be deleted.

Line 330: The year has been deleted from this phrase.

**2.19** L181: What is peak area ratio?

Line 224: The peak area ratio has been defined. In Gas Chromatography (GC), peak area ratio refers to the ratio of the area under a specific peak to another peak's area, usually that of an internal standard or another reference compound.

**2.20** L189: Keep it consistent, and use isoamyl acetate throughout the text to avoid confusion, if isoamyl acetate and isopentyl acetate are the same.

All references to isopentyl acetate have been replaced by isoamyl acetate.

**2.21** L193-195: If we take 5 microliter of stock solution and mix it with distilled water to achieve 10 ml of the desired solution, then that is only one concentration. It would be good to at least mention how many dilution steps were done.

Line 248: The text has been adapted accordingly to your suggestion.

**2.22** L197: there should be a multiplication sign here instead of a dot

Line 249: The manuscript has been adapted accordingly.

**2.23** L199-200: at this point the reader does not even know what is the experimental setup, so this sentence is a but out of context here

Line 329-424: This phrase has been moved under the training and testing procedure section.

**2.24** L219: why was the last time point different in the artificial urine compared to water?

The difference in the last time point between artificial urine and water was due to technical circumstances.

**2.25** L226: delete „in 2023”, it is mentioned after that twice

Line 330: Duplication has been removed.

**2.26** L227: what is an Algeco modular unit?

Line 331: The description of the Algeco modular unit has now been clarified in the manuscript. It refers to a type of prefabricated, portable building used for various purposes, such as offices, classrooms, or temporary accommodation. These units are modular and can be easily transported, assembled, and reconfigured to suit different needs.

**2.27** L236: there is no Figure 1C

This error has been corrected in the updated version of the manuscript.

**2.28** L243-244: How did the authors ensure that the clean water (relatively odorless) removed every residue of the isoamyl acetate solution (target scent) from the container?

Line 362: We opted for clean water because it is odorless and is expected to effectively remove any potential droplets of isoamyl acetate from the container. While some teams use alcohol/ethanol for cleaning, we believe this is not ideal, as alcohol has a strong odor that could interfere with the training, potentially causing the dogs to react to the smell. We added this information into the manuscript. In the evening a more thorough cleaning with alcohol was conducted, leaving enough time to evaporate.

**2.29** L250-251: If there were four cones and at least six trials, how did the trainers ensured that the dogs will not favor one cone for another? E.g. in case of six trials the target scent could have been in cone no 1 and 2 twice, and in cone no 3 and 4 only once, which could have increased for preference for cones no 1 and 2 in the line-up.

Line 367: The reviewer is correct, but we attempted to minimize this bias by making the target odor location pseudorandom, ensuring it was not in the same cone for more than three consecutive trials. We observed that dogs tended to prefer the first cone, likely to quickly please the handler. To address this, we plan to use a carousel configuration for the next trials. This information has been added to the manuscript.

**2.30** L254-256: how many trials were allowed at each concentration level to reach pass the criterion?

Line 376: The amount of trials was a maximum of 4 with each trial (= line) consisting of 4 cones. This information has been added to the manuscript. This information has been added to the manuscript.

**2.31** L267: Dogs could only achieve 75% success in single blind tests (2 or 3 trials), if they were correct in all trials. Right?

Line 376: The 75% success rate applies exclusively to the four trials conducted under double-blinded conditions. In the preliminary single-blinded phase, we required dogs to correctly complete two trials before advancing to the double-blinded phase. If a dog succeeded in only one out of the first two trials, a third trial was conducted to provide an additional opportunity for qualification. This clarification has been incorporated into the manuscript.

**2.32** L271: dog WAS always rewarded

Line 404: This mistake has been corrected in the updated version of the manuscript.

**2.33** L279-288: What was the reason for the weather data analysis? Based on L230, I thought the tests were done indoors in a temperature and humidity controlled room.

Line 537-540: Correct, the tests were conducted indoors. However, we were exploring the use of Algeco modular units as an environment for canine medical testing. It was important to verify if the indoor temperature and humidity were correlated with outdoor conditions. Since the dogs are from outdoors and often play outside between sessions, potentially influencing their nose capacity, working with living animals introduces numerous uncontrollable factors. Therefore, we decided to measure and check all available factors, including weather data, that could potentially influence the dogs' behavior and their ability to detect COVs. This information has been added to the updated version of the manuscript.

**2.34** L292: citation is missing for BORIS (Friard and Gamba, 2016)

Line 439: We thank the reviewer for this adjustment. The reference has been added to the manuscript.

**2.35** L305: there is an extra „s” at the end of the sentence

Line 464: This mistake has been corrected.

**2.36** L311: Is there an approval number?

Dogs used in this study were owned dogs, appropriately followed by vets to ensure an appropriate health status and mental well-being. The environment provided fulfilled all welfare requirements. Since this study is outside the scope of European regulatory frameworks, no ethical committee approval number was required.

**2.37** L315-321: The preparation of the artificial urine is already described in the methods.

Line 475: We removed this redundancy.

**2.38** L323-325: Any numbers or statistics about the significant decrease would be appreciated here.

Line 495: We have added the percentage reduction based on averages to provide clarity. However, performing statistical analysis does not seem particularly relevant in this case, as the clear decrease is evident, and the focus is on how much it decreases rather than its statistical significance.

**2.39** L326-329: Any statistical info would be appreciated here.

Line 495: We have added the percentage reduction based on averages to provide clarity. However, performing statistical analysis does not seem particularly relevant in this case, as the clear decrease is evident, and the focus is on how much it decreases rather than its statistical significance.

**2.40** L332-335: It would be easier for the reader if the results would be reported consistently in the text (either all % in brackets, or all lines in brackets).

Results are now represented consistently.

**2.41** L340-342: I don't understand this sentence. According to the methods, there were 3 dilutions used: 6.729 × 10⁻⁶ M, 6.729 × 10⁻⁷ M, and 6.729 × 10⁻⁸ M. So how could the yielded threshold be anything else for the two dogs?

Line 239-252, 254, 315: believe there may be some confusion here. Three concentrations (6.729 × 10⁻⁶ M, 6.729 × 10⁻⁷ M, and 6.729 × 10⁻⁸ M) were tested for stability in artificial urine. However, as described in the methods section (line 194), additional concentrations were prepared for threshold testing in dogs.

**2.42** L344-345: at least a p value or some numbers would be appreciated here

Line 520: P-values have been added to the updated version of the manuscript.

**2.43** L347: „Dogs marked the first cone in the line more frequently” – I don't really see this from Table 4, but what I see is that the dogs were allowed to go through the line-up only once, as they were quite likely to signal at the last cone.

There was an error in Table 4, and this has been corrected. The data now accurately reflects that the dogs were more likely to signal the first cone, knowing that they were allowed to go through the line-up only once.

**2.44** L348-349: This table is about sweeping, and the p value does not match in the text and the table.

There was an error in the name of Table 3, this has been corrected in the updated version of the manuscript.

**2.45** L352: „data not show” – Table 3 seems to contain such information?

As mentioned, the name of the table was incorrect. Table 3 represents the behavior and performance of the dogs in relation to the age of the samples.

**2.46** L353-358: The impact of weather conditions is already mentioned in the methods. I don't even know what is the point of this part, if the tests were done indoors.

Line 537: See answer 2.33. This information has been added to the manuscript.

**2.47** L370-371: why is that study less stringent, than the current study? the same principles were used to find the detection threshold by lowering the concentration of the target odor

Line 554: The study of Concha et al. (2019) is less stringent, as they set the success threshold at 40%, whereas we established it at 75%. The manuscript has been revised for clarity to reflect this distinction.

**2.48** L411-412: „with a selection of the bowl directly in front of the experimenter” – I would remove this statement, as the experimenter was not standing next to the bowl in this study; that part is not in accordance with the results. The operator was isolated in a separate part of the unit.

Line 707: We thank the reviewer for this comment. The phrase has been removed.

**2.49** L451-453: I would also add "conducting tests with medically more relevant odors".

Line 699: We include a phrase on the need of tests with medically relevant odors.

**2.50** L499: Table 1:
Wouldn't it be better to call the „No comment” the „correct search”, when the dog did everything in the way it was expected to do?
No selection: I would suggest using „dog sniffing” instead of „dog tested”, also „marking” is when a dog urinates on something therefore I would change that to „signalling”. „this comment does not apply” – This is a bit confusing remark here. The non-selection does not apply?
Sweep: So this was not allowed? The dogs had to signal on the first occasion in the correct cone?

The table has been adapted accordingly to the comments.

**2.51** L500: Table2: Be a bit more specific in the table caption, at least mention that the results of all the lines and the double-blind lines, and also stat what is in the brackets.

The table caption has been adapted accordingly to the comment.

**2.52** L501: Table 3: Based on the table caption, I would say this table is redundant, and the p values could be added to the text. However, if the authors wanted to show how the age of the samples affected the dogs' behavior and performance, then they should state it in the table caption. Further, state what is in the brackets.

The caption of Table 3 has been revised for clarity, specifying that the table addresses sample age and is therefore not redundant with the previous table. The p-values have also been added to the text, and the caption has been further adapted in response to the reviewer’s comments.

**2.53** L508: Table 5: What is the point of this table? It could be easily described in one sentence in the text.

The table 5 has been removed from the manuscript and described within the text.

**2.54** L520: Figure 1A: Based on this picture, the reader might think that the target scent was always located in the last in the line-up. More description is needed here. Further, it is confusing to see isopentyl acetate when the authors mention isoamyl acetate almost everywhere in the text. If isoamyl acetate and isopentyl acetate are the same (or can be used as synonyms) then the authors should stick to one of them to avoid confusion.

The figure caption has been revised according to the reviewers' comments. Isopentyl acetate has been replaced with isoamyl acetate, as they refer to the same compound.

**2.55** L522: Figure 2: What makes the third single-blind test optional?

Line 376: The protocol includes two trials under simple blinded conditions (SA), with an optional third trial. If the dog successfully completes the first two trials, the third trial is optional (at the handler's discretion). However, if the dog passes one trial and fails the second, a third trial is conducted to determine the outcome

**2.56** L523: Figure 3: The text is tiny on this figure, it is barley readable.

The figure has been updated as recommended in the updated version of the manuscript.

**2.57** L546: missing journal name and issue number

I did not notice any issues with the references, and they may have already been updated. Could you please specify the references you found problematic? If not, I assume this will be reviewed thoroughly if the article proceeds to the next steps for publication.

**2.58** L551, L554, L562, L601, L605, L610: missing page numbers or article number

Please, see answer 2.57.

**2.59** Supplementary Table 2: What is BDD? Is the total number of references relate to the Supplementary Table 1? Is the urine concentration range created from the lowest and highest concentration reported, or the lowest and highest reported average? In several rows in the compound name does not match the name mentioned in the description. In several descriptions it seems the beginning of the description is missing, or it begins with a half sentence, or the compound name is missing.

- BDD means database (translation from the French word) referring to the Supplementary Table 1. It has been removed herein as it is not necessary.
- The total number of references corresponds to the 12 articles reported in the Supplementary Table 1.
- The urine concentration range is created from the lowest and highest concentration reported.
- Names have been corrected to use the same denomination in both name and description columns.
- Descriptions have been rewritten in this column to correct syntax and typographical errors.

**2.60** Supplementary Table 3: In Supplementary Table 2 dots were used as decimals, while here it is a comma. Please make it consistent.

In supplementary Table 3, commas have been replaced by dots to be consistent.

**2.61** Supplementary Table 4:
Panting: OPEN mouths

This mistake has been corrected in the updated version of the manuscript.

**2.62** Is the conductor the operator?

Conductor is the handler, this wording has been adapted for consistence in the updated version of the manuscript.

**2.63** Avoiding physical contact: who is the someone who would touch the dog during testing?

The handler being present in the room, physical contact may occur at the end when the dog is rewarded (through play). This has been added to the table.

**2.64** Lowering head: lowering to what extent, how much?

We adapted an ethogram from a Bryant et al. 2018 study. The original description was: Flexion of the front and rear limbs and/or downward movement of the head to any degree without sternal contact with the floor. However, given the camera angle, it was challenging to be very precise with this observation. Therefore, we simplified the description based on what could be reliably observed. We included the following clarification in the document: Flexion of the front and rear limbs or head to any degree without sternal contact with the floor.

**2.65** Barking, whipper, backwards ears, lifts the leg while sniffing: event instead of even; lifts which leg? Why is it important?

We attempted to observe which paws were lifted; however, the camera position did not allow for sufficient precision. We only have accurate information for the first two paws. The objective of these observations was to assess the dog's emotional state. This information has been added to the manuscript.

**2.66** Play with the handler: what is real play mean? Is there unreal play interaction too? what did they do for playing?

Answer: We observed whether the play involved the handler and the dog or just the dog with its toy. In cases of play with the handler, it primarily consisted of "tug-of-war" type games. We replaced the word "real" with "spontaneous." These are play moments with the handler that were not prompted by the designer of the experiment.

**2.67** Supplementary Table 5: There is no need for this table. The information is so little that it could be just written in the text.

The Supplementary Table 5 has been removed and inserted as text within the manuscript.

**2.68** Supplementary Figure 1: Where is the figure caption? What do we see on the figure? What is peak area ratio, what is TO, what do the whiskers show?

A caption for the Supplementary Figure 1 has been added under the Figure.

For more clarity and coherence with the terms employed in the manuscript, the legend has been modified:

- “TO” is replaced by “t0”
- “TO + 7 days” is replaced by “Ratio t0+7 days/t0”

Figure 1 : Bar charts showing the relative intensities of GC-MS peak areas of VOCs in the model urine matrix at the initial time (“t0”) and after 7 days of storage at 4°C (“t0 + 7 days”). The peak areas at the initial time (“t0”) are expressed as 100% reference values (blue bars). The “Ratio t0 + 7 days/t0” (red bars) indicates the percentage ratio of values obtained after 7 days of storage to the initial values. Error bars represent the average standard deviation (n=3).

Description: The peak intensities ratio between the analysis at t0 +7 days and the initial analysis at t0 (in %) showed no significant variation for the 12 VOCs in the model urine matrix, indicating consistent signal intensity between the analysis conducted immediately after preparation (t0) and the analysis performed after 7 days of storage at 4°C.

**2.69** Supplementary Figure 2: Where is the figure caption? What do the boxplots show (lines, whiskers, diamonds shaped dots)? Where is the rest of the green boxplot, or is the bottom of it is 0? What is the measure of the area, squared meter, squared centimeter etc.? Theoretical concentration of what (x axis description)?

The boxplot displays the interquartile range, with the central line representing the median. The whiskers extend to the range of the data (1.5 IQR from the quartiles), and the diamond-shaped dots indicate outliers. The missing part of the boxplots corresponds to 0 values, which are not visible due to the log scale used in the plot. The Y-axis represents the area under the curve refers to the integrated signal corresponding to a specific compound or ion as it elutes through the chromatographic column. This area is used to quantify the amount of a particular substance in the sample. The X-axis represents the theoretical concentration of Isoamyl Acetate in water. This information has been updated in the figure description.

**2.70** Supplementary Figure 3: Same problem, as with Supplementary Figure 2

The boxplot displays the interquartile range, with the central line representing the median. The whiskers extend to the range of the data (1.5 IQR from the quartiles), and the diamond-shaped dots indicate outliers. The missing part of the boxplots corresponds to 0 values, which are not visible due to the log scale used in the plot. The Y-axis represents the relative response in GC-MS, which is the intensity of the analyte signal relative to the total ion current or to an internal standard, allowing for comparison across different concentrations and conditions. The X-axis represents the theoretical concentration of Isoamyl Acetate in water. This information has been updated in the figure description.

6. PLOS authors have the option to publish the peer review history of their article ([what does this mean?](https://track.editorialmanager.com/CL0/https:%2F%2Fjournals.plos.org%2Fplosone%2Fs%2Feditorial-and-peer-review-process%23loc-peer-review-history/1/010f0193bf50fa05-6bc64c74-d5ab-4d63-a44e-b1b3571f2d93-000000/ILm1BqV8YBfW1-mg7hZqFsXIJTXpwe6Hex9Xw4KQotk=189)). If published, this will include your full peer review and any attached files.


If you choose “no”, your identity will remain anonymous but your review may still be made public.


**Do you want your identity to be public for this peer review?** For information about this choice, including consent withdrawal, please see our [Privacy Policy](https://track.editorialmanager.com/CL0/https:%2F%2Fwww.plos.org%2Fprivacy-policy/1/010f0193bf50fa05-6bc64c74-d5ab-4d63-a44e-b1b3571f2d93-000000/n813udOlIvSwr0YsVoNqQI7FbFxZRxlyXM___fQmOh0=189).

Reviewer #1: No

Reviewer #2: No

While revising your submission, please upload your figure files to the Preflight Analysis and Conversion Engine (PACE) digital diagnostic tool, [https://pacev2.apexcovantage.com/](https://track.editorialmanager.com/CL0/https:%2F%2Fpacev2.apexcovantage.com%2F/1/010f0193bf50fa05-6bc64c74-d5ab-4d63-a44e-b1b3571f2d93-000000/3rGDR26wQDw8wrdxsaLJWYDHlpeGwm0Wat2druTo9_I=189). PACE helps ensure that figures meet PLOS requirements. To use PACE, you must first register as a user. Registration is free. Then, login and navigate to the UPLOAD tab, where you will find detailed instructions on how to use the tool. If you encounter any issues or have any questions when using PACE, please email PLOS at [figures@plos.org](mailto:figures@plos.org). Please note that Supporting Information files do not need this step.
